# Controller and battery changes due to technical problems related to the HVAD® left ventricular assist device - a single center experience

**DOI:** 10.1186/s13019-018-0759-9

**Published:** 2018-06-19

**Authors:** Emil Najjar, Ann Hallberg Kristensen, Tonje Thorvaldsen, Laila Hubbert, Peter Svenarud, Magnus Dalén, Agneta Månsson Broberg, Lars H. Lund

**Affiliations:** 1 Department of Medicine, Unit of Cardiology, Karolinska Institutet, Karolinska University Hospital, S3:02, Stockholm, 17176 Sweden; 2 Heart and Vascular Theme, Karolinska University Hospital, Stockholm, Sweden; 3 Department of Molecular Medicine and Surgery, Karolinska Institutet, Stockholm, Sweden

**Keywords:** Heart failure, HVAD®, Left ventricular assist device, Alarm, Device malfunction

## Abstract

**Background:**

The use of left ventricular assist devices (LVADs) has increased in the last decade. Major complications have been well described, but there is no data on device alarms and actual or threatening malfunction which impair quality of life and may impair outcomes. This study describes the technical problems related to the use of the HVAD® left ventricular assist device in a single center.

**Methods:**

We retrospectively reviewed device malfunctions and outcomes in 22 patients with HVAD**®** left ventricular assist device followed at Karolinska University Hospital between 2011 and 2016. Device malfunction was defined by INTERMACS as a failure of one or more of the components of the LVAD system. The primary outcome was defined as death or hospitalization or unplanned urgent clinic visit due to device alarm of unknown significance or actual or threatening malfunction. Separate secondary outcomes were malfunction resulting in controller exchange and malfunction resulting in battery change. Exploratory outcomes were death, transplantation, or explantation because of recovery.

**Results:**

Median age was 59 years and 19% were women. Over a mean follow-up time of 1.7 years (37 patient-years), the primary outcome occurred 30 times (0.8 events per patient-year; 0 deaths, 2 hospitalizations and 28 un-planned clinic visits). Secondary outcomes were 41 device malfunctions for 14 patients requiring 45 controller exchanges in 12 patients (1.1 events per patient-year) and 128 battery changes in 12 patients (3.5 events per patient-year). Exploratory outcomes were 8 deaths (36.4%), 7 transplantations (31.8%) and 2 explants due to recovery (9.1%).

**Conclusion:**

The use of HVAD® was associated with technical problems requiring frequent un-planned clinic visits and changes of controller and/or batteries. There were no deaths due to device malfunction. Further studies are warranted to evaluate the risk of device malfunction and associated reductions in quality of life and cost.

## Background

Heart failure (HF) is associated with impaired quality of life, poor prognosis and high costs to society [1–3]. Heart transplantation is still the best available treatment for end-stage HF for appropriate patients, but it is restricted by the limited number of donor organs. This organ shortage has led to increased use of left ventricular assist devices (LVADs) during the last decade [4, 5] as bridge to transplantation (BTT) [6, 7] or destination therapy (DT) [8].

Despite advances in technology, LVADs are still associated with complications such as infection, bleeding, thrombosis and stroke, which have been well described in the literature. Furthermore, device malfunction may be life threatening [9–11]. Therefore, algorithms and alarms designed to detect or avert threatening or actual device malfunction have been developed and are incorporated into device controller units. The HeartWare**®** left ventricular assist device (HVAD**®**; HeartWare*®*, Inc. Framingham, MA, USA) is a small intrapericardial centrifugal-flow LVAD approved by the United States Food and Drug Administration (FDA) as a bridge to transplantation in 2012 [12]. Anecdotally, we observed frequent device alarms and malfunction in our LVAD clinic. Even though they may not represent or cause serious clinical complications, device alarms and malfunction can be a major nuisance, cause anxiety and reduce quality of life for patients, and increase the burden and cost for health care providers for device checks and potential related care. These have not previously been previously quantified. We systematically reviewed our experience with HVAD**®**-related device malfunctions.

## Methods

### Patients

All patients followed at the Karolinska University Hospital after HVAD*®* implantation between 2011 and 2016 were reviewed retrospectively. The retrospective review of patient outcomes had ethics approval; individual patient consent was not required or obtained.

Baseline and outcome data were abstracted from the patient medical records. In the clinical setting these patients are seen every 1–2 weeks in an out-patient nurse based LVAD-coordinator clinic which includes documentation of LVAD settings, readouts and alarms. All hospitalizations for patients with an LVAD occur at our hospital. In addition, we had access to all HVAD*®* log files saved due to technical problems during the study period.

A device malfunction was defined according to INTERMACS as a failure of one or more of the components of the HeartWare**®** system [13].

### Outcomes

All outcomes were presented in the entire cohort as number of events per patient-year (EPPY). The primary outcome was defined as death or hospitalization or unplanned urgent (within 12 h of the patient’s call to our center to report a device alarm) clinic visit due to device alarm of unknown significance or actual or threatening malfunction. The total number of controller exchange/battery change was displayed in a bar chart for each patient. Separate secondary outcomes were malfunction resulting in controller exchange (which temporarily stops the pump and carries risk) and malfunction resulting in battery change. Each controller and battery exchange was recommended by HeartWare*®*, Inc. support staff. Exploratory outcomes were death, transplantation, or explantation because of recovery.

### Statistical analysis

Statistical analysis was performed using SPSS version 23.0 (SPSS Inc., Chicago, Ill. USA). Baseline continuous variables are expressed as median (interquartile range) and compared by the Mann-Whitney’s test while categorical variables are presented as numbers and percentages and compared using Fischer’s exact test.

## Results

Twenty two patients with HVAD® were included in the study (The total number of implantations at our center during the study period was 21 and all of them received HVAD® while one patient was operated in India). At time of implantation, median age was 59 (51; 65) years, 19% were women, median body surface area (BSA) was 1.96 (1.76; 2.10) m^2^, median estimated glomerular filtration rate (eGFR) was 56 (38; 78) mL/min/1.73 m^2^ and median EF was 20% (15%; 25%). Idiopathic dilated cardiomyopathy was the main underlying cause of HF and accounted for 46% of all causes. The indications for HVAD**®** were DT (32%), BTT (36%), and bridge to decision (BTD) (32%).

Table 1 details the baseline characteristics of the study patients at the time of implantation, comparing those who did (*n* = 15) vs. did not (*n* = 7) experience any device malfunction during follow-up.

**Table 1:** Patient characteristics at the time of implantation (*n* = 22)

Variable	Patients with device malfunction *n* = 15	Patients without device malfunction *n* = 7	*p*-value
Age (years)	56 (50; 68)	64 (61; 64)	0.45
Gender (female)	3 (20)	1 (14%)	0.62
BSA (m^2^)	1.96 (1.76; 2.06)	1.95 (1.73; 2.22)	0.54
eGFR (mL/min/1.73 m^2^)	61 (51; 80)	36 (29; 44)	0.001
EF (%)	15 (15; 20)	20 (15;30)	0.16
Type of cardiomyopathy:			0.44
Idiopathic	6 (40)	4 (57)	
Ischemic	7 (46)	2 (29)	
Drug/alcohol abuse	1 (7)	1 (14)	
Other causes	1 (7)	0	
Indication for HVAD® therapy:			0.44
DT	5 (34)	2 (29)	
BTT	2 (13)	2 (29)	
BTD	6 (40)	1 (14)	
BTD/DT	0	1 (14)	
BTD/BTT	2 (13)	1 (14)	

The baseline characteristics of the study patients at the time of implantation, comparing those who did (*n* = 15) vs. did not (*n* = 7) experience any device malfunction during follow-up

*Abbreviations:* *HVAD®* HeartWare**®** left ventricular assost system, *BSA* Body surface area, *eGFR* Estimated glomerular filtration rate using the CKD-EPI (chronic kidney disease epidemiology collaboration), *DT* Destination therapy, *BTT* = Bridge to transplantation, *BTD* Bridge to decision, *EF* Ejection fraction

Data are presented as median (interquartile range) or n (%)

Over a mean follow-up of 1.7 years (Total follow-up time 37 patient- years), the primary outcome occurred 30 times (0.8 times per patient-year; 0 deaths, 2 hospitalizations and 28 un-planned clinic visits). Seven of the 22 patients were transplanted, 2 had the device removed after myocardial recovery, 8 died, while 5 remained on HVAD® support at the end of the study period. Table 2 shows the outcomes in patients with and without device malfunction.

**Table 2:** Duration of support and exploratory outcomes in patients with and without device malfunctions

Outcome	With device malfunction (*n* = 15)	Without device malfunction (*n* = 7)	Duration of support (Days) overall
Dead	3 (20%)	5 (71%)	96.5 (30.5;472.3)
Remained on device	5 (33.5%)	0	1037 (350.5;1772)
Transplanted	5 (33.5%)	2 (29%)	380 (336;754)
Weaned after recovery	2 (13%)	0	709.5

Duration of support is presented as median (interquartile range)

In total; 8 patients died during the study time but only 5 patients died within 1 year after LVAD operation (2 had LVAD as BTD, 2 as BTT and the 5th patient as a bridge to decision). All those 5 patients were in INTERMACS 1 or 2 when they were implanted. They died either as a result of a multiorgan failure or secondary to stroke. Those 5 patients did not have a device malfunction during the support time.

Few cases of pump thrombosis occurred during the study time and they were all successfully treated medically using thrombolysis. There were no deaths due to pump thrombosis.

**Fig. 1:**
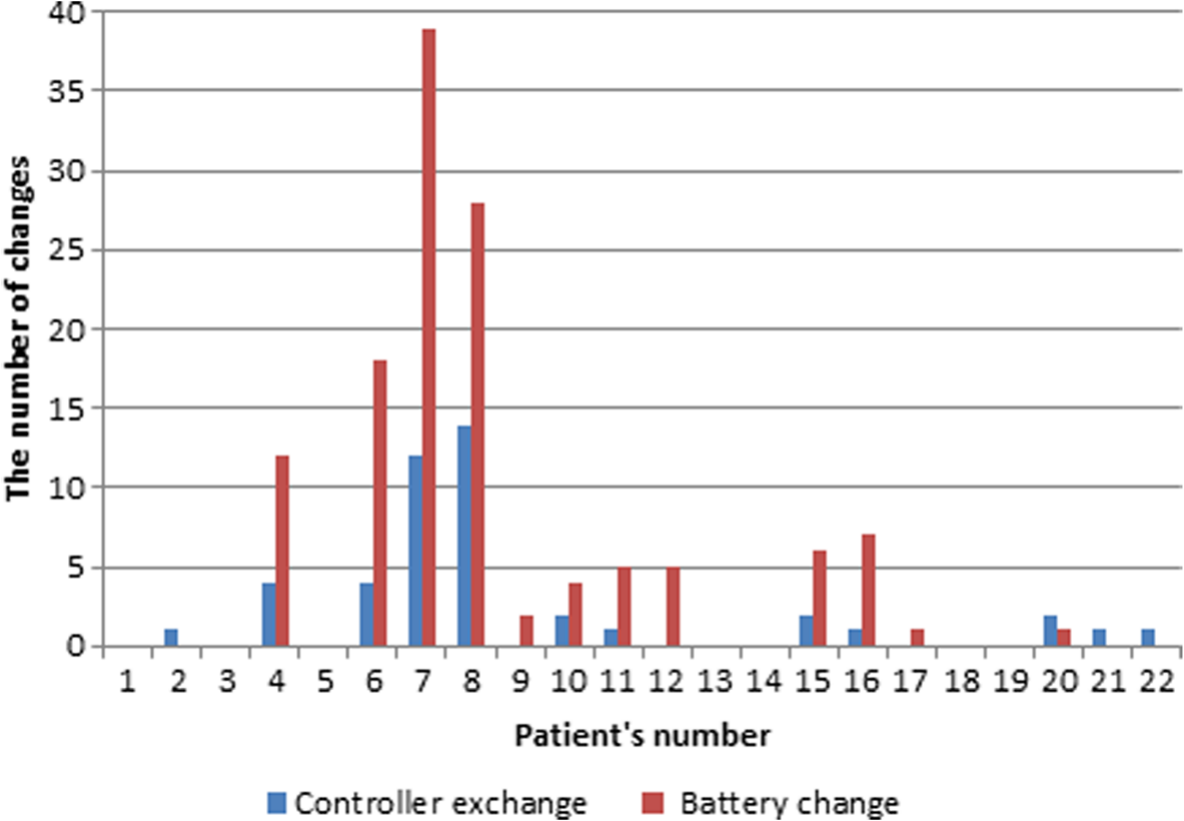
Change of controller and batteries (among *n* = 22 patients)

For secondary outcomes, 41 device malfunctions occurred (1.1 EPPY) requiring 45 controller exchanges in 12 patients (1.2 EPPY) and 128 battery changes in 12 patients (3.5 EPPY) (Fig. 1). Figure 1 illustrates that 7 patients out of 22 did not have a device malfunction.

**Fig. 2:**
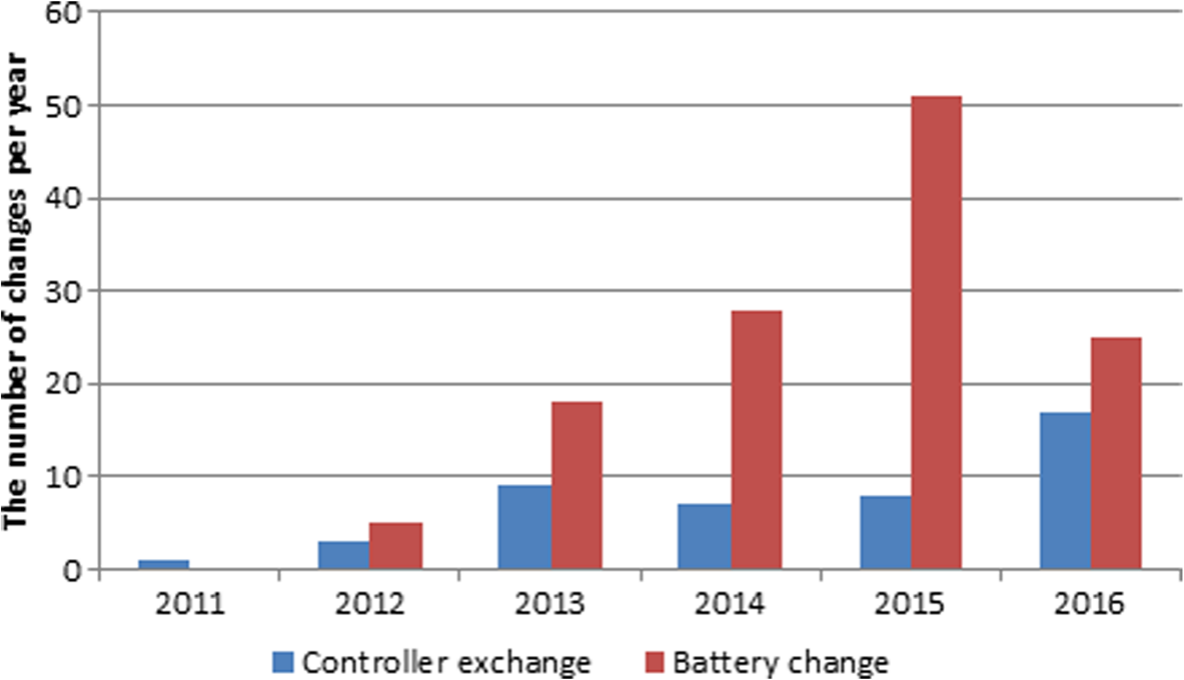
Number of changes per year (among *n* = 22 patients)

The number of controller and battery changes per year is illustrated in Fig. 2 while the definition of underlying technical problems, their frequency and the performed measures are presented in Table 3. The most common alarms were security messages and beeping without apparent reason, however, the latter required more repeated controller and/or battery changes than any other alarms. We measured blood pressure (not in all patients) using Doppler when the patients attended to our center because of alarms and there was no association between blood pressure (as a surrogate measure of after-load) and alarms; i.e. many of those who had the alarms did have a normal blood pressure.

**Table 3:** Technical problems and the needed measures performed at the same or different occasion

Technical problem	Number of events (*n* = 41)	Controller exchange (*n* = 45)	Batteries change (*n* = 128)
Beeping without reason	7	18	82
Security message	10	9	5
Sudden change in charge capacity of a battery	3	1	5
Red alarm (black display)	3	3	1
The controller switches back and forth between batteries	3	2	8
Red flashing in battery charger	3	0	7
Critical battery	1	0	3
HVAD stopped suddenly	2	3	1
Electrical fault	2	1	0
Other reasons: loose connection, damage, no communication to monitor	7	8	16

## Discussion

To our knowledge, this is the first report to assess specifically HVAD®-related technical problems. The technical problems faced in our center did not lead to death, pump exchange or any serious consequences; however, the exchange of controller exposed the patients to high risks related to controller disconnection and the time needed to reconnect the new controller during which the patients had no pump support. Moreover, the frequent alarms impaired quality of life for patients and caused many un-planned visits with increased costs and burden for health care providers.

The current study focuses on minor technical problems which although minor, were considerably greater in incidence than any kind of technical problems or complications shown in previous studies. Previous data report only major complications, often requiring device replacement, which we had none. The first large HVAD® study, ADVANCE [6], with 140 patients, reported only 26 device malfunctions from 20 patients and 6 of these malfunctions led to a device replacement. The recent ENDURANCE reported 124 events (0.30 EPPY) of device malfunction or failure in 93 patients (31.4% of the study group); however, there were no details about the cause or the type of device malfunction or failure [14]. Additionally, 2 HVAD® studies which included 242 patients reported only 10 cases of manufacturing defect or device exchange due to other causes without specifying the cause [9, 15]. The post-marketing registry study (ReVOLVE) did not report any case of technical problems [12].

Addtionally, a systematic search of PubMed and Web of Science did not reveal any reports similar to ours. However, HeartWare® Inc. recently sent an urgent medical device correction letter to recall HVAD® controllers of some models due to a loose connector that may allow moisture to enter the controller with increased risk of corrosion, electrical issues, reduced speaker volume and connection failures. Another safety alert was sent by HeartWare® Inc. in June 2016 to recall the batteries of HVAD® with specific serial numbers because they may lose power prematurely due to faulty cells [16, 17].

The software of all controllers was upgraded at our center in May 2016 but the problems with malfunctions continued. All technical problems were solved according to the recommendations of the manufacturer by changing the controller or the batteries. Two out of the 4 patients who had the highest percentage of alarms continued to have frequent alarms despite the implemented procedures. The first one continued to have alarms until death and the second one until transplantation. The other 2 patients experienced fewer alarms after implementing the recommended procedures and software upgrading. The medical staff taking care of our HVAD® patients is experienced with different types of ventricular assist devices and our patients received the required education needed to handle a mechanical pump so the question that remains unanswered is the underlying cause that can explain these technical problems.

Furthermore, we do not know whether our center is unique regarding this issue or whether the problem is underreported by other hospitals.

### Limitations

The study is limited by the small sample size and being a single center study. The findings need confirmation by other studies.

## Conclusions

The use of HVAD® in our center was associated with a high rate of technical problems requiring frequent un-planned clinic visits and changes of controller and/or batteries. There were no deaths due to device malfunction. Further studies are warranted to study the risk of device malfunction and associated reductions in quality of life and cost.

## Abbreviations

BTD: Bridge to decision
BTT: Bridge to transplantation
DT: Destination therapy
eGFR: Estimated glomerular filtration rate
EPPY: Events per patient-year
FDA: the United States Food and Drug Administration
HF: Heart failure
HVAD®: HeartWare®HeartWare® Left ventricular assist device
LVADs: left ventricular assist devices
